# A Role for B Cells to Transmit Hepatitis C Virus Infection

**DOI:** 10.3389/fimmu.2021.775098

**Published:** 2021-12-16

**Authors:** Isabelle Desombere, Freya Van Houtte, Ali Farhoudi, Lieven Verhoye, Caroline Buysschaert, Yvonne Gijbels, Sibyl Couvent, Wilfried Swinnen, Hans Van Vlierberghe, André Elewaut, Andrea Magri, Zania Stamataki, Philip Meuleman, Jane A McKeating, Geert Leroux-Roels

**Affiliations:** ^1^ Department of Diagnostic Sciences, Ghent University, Ghent, Belgium; ^2^ Medisch Sociaal Opvangcentrum (MSOC) Gent, Ghent, Belgium; ^3^ Department of Hepatology and Gastroenterology, Ghent University Hospital, Ghent, Belgium; ^4^ Laboratory of Hepatology Research, Ghent University, Ghent, Belgium; ^5^ Nuffield Department of Medicine, University of Oxford, Oxford, United Kingdom; ^6^ Institute of Immunology and Immunotherapy, Centre for Liver and Gastrointestinal Research, University of Birmingham, Birmingham, United Kingdom; ^7^ National Institute for Health Researc (NIHR) Birmingham Liver Biomedical Research Centre, University Hospitals Birmingham National Health Service (NHS) Foundation Trust, Birmingham, United Kingdom

**Keywords:** hepatitis C virus, B cell, transmission, infection, persistence, immune response

## Abstract

Hepatitis C virus (HCV) is highly variable and transmits through infected blood to establish a chronic liver infection in the majority of patients. Our knowledge on the infectivity of clinical HCV strains is hampered by the lack of *in vitro* cell culture systems that support efficient viral replication. We and others have reported that HCV can associate with and infect immune cells and may thereby evade host immune surveillance and elimination. To evaluate whether B cells play a role in HCV transmission, we assessed the ability of B cells and sera from recent (<2 years) or chronic (≥ 2 years) HCV patients to infect humanized liver chimeric mice. HCV was transmitted by B cells from chronic infected patients whereas the sera were non-infectious. In contrast, B cells from recently infected patients failed to transmit HCV to the mice, whereas all serum samples were infectious. We observed an association between circulating anti-glycoprotein E1E2 antibodies and B cell HCV transmission. Taken together, our studies provide evidence for HCV transmission by B cells, findings that have clinical implications for prophylactic and therapeutic antibody-based vaccine design.

## Introduction

Hepatitis C virus (HCV) has infected an estimated 70 million people worldwide and is a major cause of hepatitis, cirrhosis, and hepatocellular carcinoma (HCC) (1). The development of highly effective antiviral agents has transformed the treatment of this chronic disease (2–4), enabling curative therapies for many patients (5). However, the cost of such treatments limits accessibility in endemic regions and HCV continues to pose a major public health burden with cured patients at ongoing risk of developing HCC and worldwide the number of hepatitis-related deaths is increasing (6, 7). In response to this, the WHO published strategies for global eradication of hepatitis viruses by 2030 (8). Successful elimination will rely on curing infected patients and preventive measures to reduce the rate of new infections, highlighting the urgent need for a vaccine. The high incidence of new infections in injecting drug users (IDU) and those accidentally exposed to infected blood, justifies the continued search for a prophylactic vaccine (9–11).

HCV is a positive-strand RNA virus in the hepacivirus genus of the *Flaviviridae* family and a blood-borne pathogen. HCV replicates *via* an error-prone RNA-dependent RNA polymerase and persists as a diverse quasispecies (QS) within infected individuals (12–14). Our understanding of the correlates that associate with a protective immune response is limited (15, 16). Approximately 25% of individuals acutely infected with HCV will spontaneously clear their infection (17) and several studies have reported an association between clearance and the early development of neutralizing activity in the plasma (18, 19). Broadly neutralizing HCV-specific antibodies (bnAbs) targeting the envelope E1E2 glycoproteins can be generated during natural infection (20–23), however, they often develop late, after envelope glycoprotein mutations have given rise to new variants that escape immune detection (13, 24). Our limited ability to propagate clinical isolates of HCV *in vitro* has limited our understanding of the role antibodies play in neutralizing virus infection (25–27). HCV RNA is routinely measured in the plasma as a surrogate marker of viremia and yet the infectivity of these viral genomes is unknown.

Hepatocytes within the liver are the major reservoir supporting HCV replication, however extra-hepatic manifestations of disease have been reported (28, 29). There is ample epidemiologic evidence for an association of chronic HCV infection with B cell lymphoproliferative disorders such as mixed cryoglobulinemia (MC) and B cell non-Hodgkin lymphoma (B-NHL), both characterized by clonal expansions of B cells (30). The association between successful antiviral therapy and effective cure with an improved outcome of HCV-positive B-NHL, demonstrates the pathogenic role of HCV (31–33). HCV RNA was reported to associate with B cells and several studies have detected the presence of negative strand viral RNA and/or proteins in B cells from chronic infected patients (34–38). However, there is limited evidence of productive viral replication in these immune cells and their capacity to transmit HCV has not been explored. We previously reported (39) that the JFH-1 strain of HCV associated with CD40L-stimulated B cells and, although there was no evidence of virus replication in B cells, the complex was infectious for hepatoma cells. This led us to conclude that B cells can capture HCV and trans-infect liver cells, however, the relevance of these observations for the infectivity of B cells from chronic HCV patients has not been studied.

Since there are no fully immunocompetent small-animal models that support HCV infection, we used a well characterized immunodeficient murine xenograft uPA model (40–42) to investigate the role of B cells in HCV infection. We evaluated the infectivity and genetic diversity of HCV in peripheral blood-derived B cells or sera from a cohort of recent or chronic HCV infected patients in this model. Sera from recently infected patients transmitted HCV in the mouse model, whereas B cells from the same donors were non-infectious. In chronic infection, we observed the inverse, in that B cells successfully infected the mice and sera did not. Our experiments demonstrate a protective role for Abs in limiting cell-free infection during chronic disease and reveal a previously unrecognized role for B cells in HCV infection.

## Materials and Methods

### Patients

Fifteen (15) patients with ongoing HCV-infection were selected for inoculation experiments in chimeric uPA-SCID mice. Samples were taken early after initial HCV-infection or during persistent infection (ranging from 3 months to 30 years post exposure). HCV-genotyping of the human sera was performed using a Line Probe Assay developed by Innogenetics (Versant^®^ HCV genotype Assay). All patients were vaccinated with HBsAg and non-infected with HIV. Demographic, viral and immune characteristics of these 15 patients are summarized in **Table 1**. Samples from an additional fourteen (14) HCV patients, selected and analyzed in the same manner, have not been used in inoculation experiments. Demographic, viral and immune characteristics of these 14 patients are summarized in **Supplementary Table 1**. On average 54 mL venous blood was drawn from each patient at the doctor’s office: 9mL coagulated blood to prepare serum and 45 mL heparinized blood to isolate peripheral blood mononuclear cells (PBMC). Patients P05, P08 and P09 donated more blood for additional experiments.

**Table 1:** Demographic, virologic and immunologic characteristics of HCV-infected patients.

Patient			HCV-infection		HCV-RNA		HCV-specific IgG	
	ID	age, gender	route^a^	duration^b^	serum	B-cells	anti-E1E2_H77_^c^	neutralization to HCVpp-H77 (IC_50_)^d^
					(IU/ml)	(IU/10^6^ B-cells)		
**gt1a**	**P01**	30, M	IDU	1y	6,74 10^6^	<46	< 10	< 50%
	**P02**	28, F	IDU	≤ 1y	1,42 10^6^	<46	< 10	< 50%
	**P32**	36, F	IDU	5m	1,29 10^7^	3,51 10^4^	559	< 50%
	**P03**	34, M	IDU	1y 4m	5,30 10^6^	9,54 10^4^	4898	4065
	**P05^e^**	47, F	IDU	25y	3,70 10^6^	2,15 10^4^	90991	3311
	**P15_2009^f^**	28, M	IDU	3y	5,00 10^6^	1,11 10^5^	30440	21616
**gt1b**	**P06**	64, M	transfusion	>10y	1,25 10^7^	5,60 10^4^	259	< 50%
	**P08^e^**	68, F	transfusion	30y	2,18 10^6^	8,28 10^4^	30339	14286
	**P09**	64, M	transfusion	15y	8,91 10^5^	1,91 10^4^	3040	1858
	**P10**	47, M	transfusion	28y	1,40 10^6^	6,52 10^3^	35727	6250
	**P11**	66, M	transfusion	20y	2,02 10^6^	6,46 10^3^	4046	922
	**P12^e^**	28, F	IDU	≥ 5y	3,74 10^5^	4,31 10^3^	16218	6361
**gt3a**	**P13**	26, M	IDU	1y	5,41 10^5^	<46	< 10	< 50%
	**P14**	33, M	IDU	3m	2,88 10^6^	2,42 10^4^	109	< 50%
	**P15_2006^f^**	25, M	IDU	4m	2,29 10^7^	1,46 10^5^	165	< 50%
	**P16**	31, M	IDU	≥ 5y	9,76 10^5^	3,38 10^4^	3396	513

a IDU, injecting drug use. ^b^Time elapsed between initial HCV-infection/diagnosis and biological sampling for the present study (y, year; m, month). ^c^Anti-E1E2_H77_ IgG levels are expressed as reciprocal endpoint dilution titer of autologous serum in EIA; <10, IgG was not detected at serum-dilution 1/10. ^d^Neutralizing activities of autologous serum to HCV-pseudoviral particles expressing envelope proteins of gt1a (HCVpp_H77) are expressed as IC_50_ (reciprocal titer); <50%, neutralization did not reach 50%. ^e^Serum and B cells from patients P05, P08 and P12 were sampled at multiple time points, only 1 time-point is shown. ^f^Patient P15, initially infected with gt3a, experienced a HCV-super-infection with gt1a and has donated biological specimens twice with a 3-year interval.

### Purification of Polyclonal IgG From Human Plasma Samples

Plasma samples, obtained from HCV-infected patients, were heat inactivated (56°C for 30 minutes) and IgG was purified with a HiTrap Protein G column (GE Healthcare), as described previously (43).

### Generation and Inoculation of Hu-liv-uPA-SCID Mice

**Figure 1:**
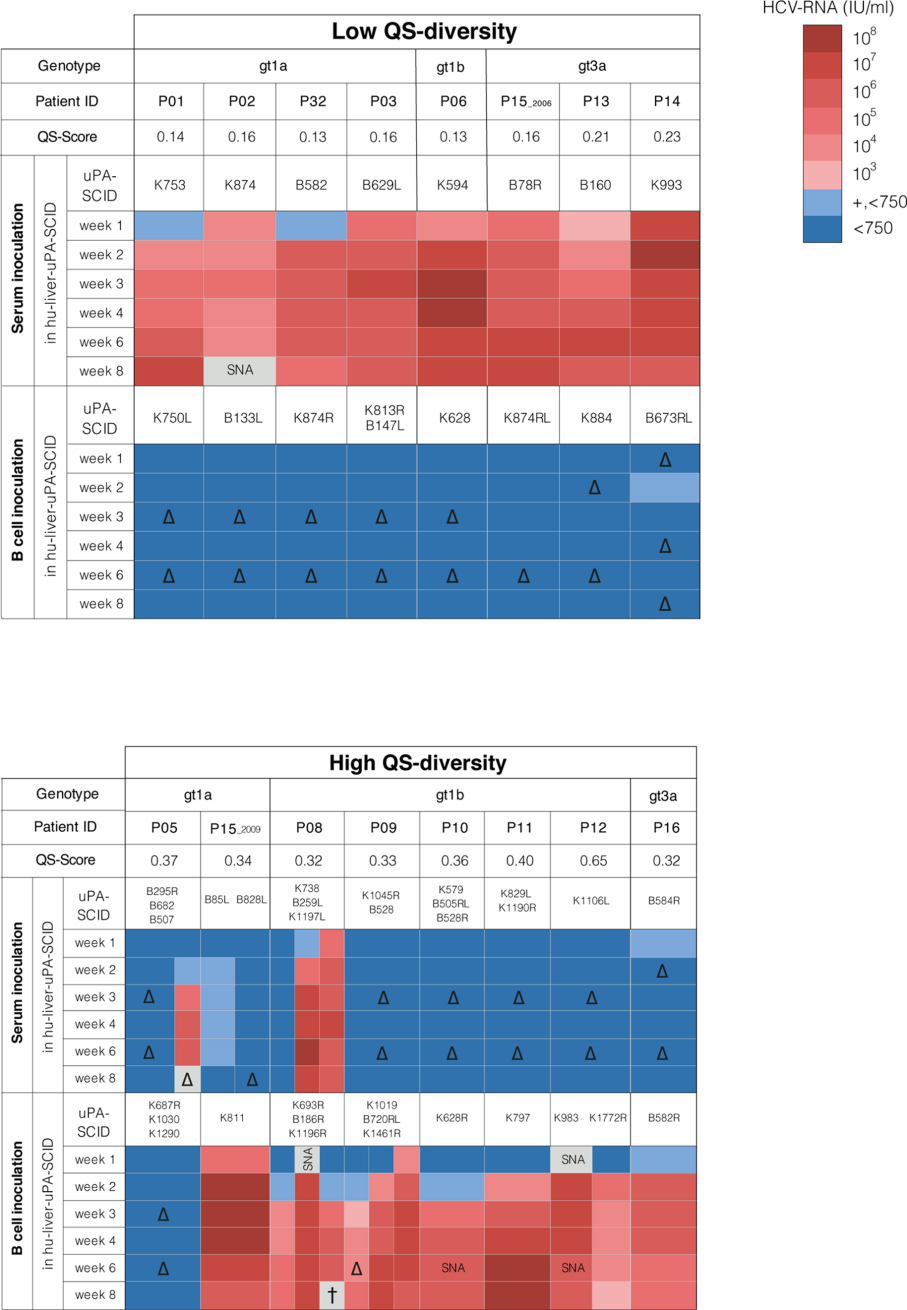
HCV quasispecies and infectivity of serum or B cells in hu-liver-uPA-SCID mice. HCV-infected patients (gt1a, gt1b and gt3a) are grouped according to their E1E2 quasispecies (QS) diversity and QS-score defined by maximum-likelihood phylogenetic trees of the E1E2 region (**Supplementary Figure 2**). Patients were grouped by low QS-score <0.25 (recent infection) and high QS-score >0.30 (chronic infection). Serum containing 3x10^5^ IU HCV RNA was injected into the peritoneal cavity or 10^6^ B cells delivered *via* the intra-splenic route. Mice were bled 1, 2, 3, 4, 6 and 8 weeks post-inoculation and plasma analyzed for HCV RNA level that are indicated by a heat map. ‘+, <750’ means that HCV RNA was detectable but <750 IU/ml (LOQ). Repeated experiments are indicated by hu-liver-uPA-SCID mice numbers. Patient P15 experienced a HCV super-infection and donated blood in 2006 and 2009. SNA, sample not available. ∆, not done (color code extrapolated). †, animal died before week 8.

Breeding and genotyping of *alb*/uPA-CBySmn.CB-17 Prkdc^scid^ (uPA-SCID) mice have been described previously (41, 44). In brief, two-week old SCID mice of both sexes, homozygous for the uPA-transgene, were transplanted with cryopreserved human hepatocytes from a single HCV-uninfected donor as described previously (41). Five weeks after transplantation, the extent of liver humanization was assessed by quantifying the concentration of human albumin in mouse plasma. Only animals with a high repopulation grade, defined by a plasma human albumin (hu-alb) content of 1 mg/mL or greater, were used. Plasma albumin levels of all mice used in the different experiments ranged between 2 and 10 mg/mL (**Supplementary Table 2**). Hu-alb levels were quantified with the Human Albumin ELISA Quantitation kit (Bethyl Laboratories Inc.). Six weeks after hepatocyte transplantation, inocula consisting of infected serum or plasma were injected in the peritoneal cavity of human liver-uPA^+/+^SCID mice (IP). Purified B cell suspensions were injected in the spleen under visual control (IS) in the same manner as human hepatocytes had been injected before to establish chimerism. Unlike viral particles or proteins present in serum or plasma that can move unhindered from the peritoneal cavity *via* the general circulation to the liver, cellular components like hepatocytes or B cells need to be injected directly into the spleen in order to reach the liver *via* the portal vein. Animals were sampled weekly and followed until 8 weeks after infection. Mouse plasma samples were stored at -80°C until analysis. One to three chimeric mice were injected with serum/plasma or B cells from each of the 15 HCV patients. Each individual mouse was given a unique number that is shown in **Figure 1** and **Supplementary Table 2**.

### Isolation of Human B Cells

PBMC were isolated from whole heparinized blood by density gradient centrifugation. Human B cells were positively selected from PBMC using the MACS^®^ Column Technology (Miltenyi Biotec). In brief, anti-CD19 coated microbeads were incubated with PBMC for 15 min at 4°C. After washing, the cell suspension was loaded onto the prepared MS Column. The unlabeled cells run through while the magnetically labeled cells are retained on the column. After washing, the column is removed from the magnetic field and the magnetically retained cells can be flushed out as the positively selected cell fraction. All CD19^+^ B cell populations used in these experiments met the set criteria of purity (≥95% required) and viability (≥85% required) as measured using flow cytometry.

### RNA Extraction for In-House RT-PCR Amplifications

RNA extraction from serum was done using the High Pure Viral Nucleic Acid Kit (Roche Diagnostics). For total RNA-extraction from B cells we used the RNeasy Mini Kit (Qiagen). Since B cells could be lost during washing steps, the number of B cells was normalized to the amount of total RNA after extraction (NanoDrop, Thermo Fisher Scientific).

### HCV RNA Quantification

HCV RNA levels from human serum or mouse plasma were quantified using the COBAS Ampliprep TaqMan HCV Assay (Roche Diagnostics). Mouse plasma samples were tested at a 1/50 dilution, resulting in a limit of quantification (LOQ) of 750 IU/ml. The HCV RNA content of B cells was measured after total RNA extraction from 5x10^5^ B cells. cDNA was synthesized using Superscript™ III Reverse Transcriptase (Invitrogen, Life Technologies) with random primers according to standard procedures. Five microliters of cDNA were used for HCV RNA quantification with the COBAS TaqMan HCV Assay, resulting in a LOQ of 46 IU HCV RNA/10^6^ B cells. All extractions and amplifications were run according to universally adopted precautions, such as the use of different rooms for pre-PCR experiments, in order to avoid cross-contamination.

### Quasispecies Analysis: E1E2 Amplification, Plasmid Generation and Sequencing

Quasispecies analysis based on sequencing of the envelope proteins was performed on HCV recovered from human serum, B cells and uPA-SCID plasma. After RNA-extraction, cDNA was synthesized as described and the HCV envelope region was amplified by means of a nested PCR with envelope-specific primers (45). Full-length E1E2 PCR products (containing EcoRV cleavage sites) were purified and cloned in the bacterial pCR^®^-Blunt vector (Zero Blunt^®^ PCR Cloning Kit, Invitrogen). After plating of transformed cells and plasmid isolation (QIA prep Spin Miniprep Kit, Qiagen), the E1E2 region of the different clones was sequenced using the SANGER technique (ABI3730XL DNA Sequencing System; Applied Biosystems). Amino acid (AA) sequences were deduced and aligned using CLC-DNA workbench (v5.7) and BioEdit Sequence Alignment Editor. Visual inspection of the chromatograms confirmed AA diversity. Maximum-Likelihood phylogenetic trees showing the relationship between E1E2_192-745_ sequences were inferred under the LG model (46) of AA substitution with estimated γ-distribution parameter, using PhyML software (47) (v3.0; South of France bioinformatics platform; ATGC). The tree topology (starting with a Neighbor-Joining tree) was optimized using the Nearest Neighbor Interchange (NNI) approach. Bootstrapping with 1000 replicates tested the statistical robustness of the phylogenetic trees. Only branching with a bootstrap value of ≥ 70% was defined as robust clustering.

### Detection of (-) and (+) Sense HCV RNA by Tth Reverse Transcriptase PCR

For strand-specific HCV-RNA detection, the Tth-based method with minor modifications and appropriate controls was used (48–50). After total RNA isolation from B cells, RNA was denatured (95°C for 5 min) and RT-PCR was performed using a thermostable Tth DNA Polymerase/Reverse Transcriptase (Promega). Positive strand-specific cDNA was generated using an antisense (5’-CACTCGCAAGCACCCTATCA-3’; 5UTR-6-R) primer and negative strand-specific cDNA using a sense (5′-CTGTCTTCACGCAGAAAGCG-3′; 5UTR-1-F) primer for reverse transcription. Strand-specific cDNA was amplified by adding the other primer to target a 256bp region of the 5’ non-coding region. Twenty cycles (94°C for 30s; 60°C for 30s; 72°C for 30s) were run, followed by 72°C for 7 min. After initial denaturation (94°C for 2 min), nested PCR with Taq DNA Polymerase (NEB) and sense and antisense primers (5’-AGCGTCTAGCCATGGCGT-3’; 5UTR-2-F and 5’-CTCGGCTAGCAGTCTTGCGG-3’; 5UTR-5-R) was performed using 35 cycles (94°C for 30s; 60°C for 45s; 68°C for 30s), followed by a last extension at 68°C for 5 min. Amplified products (185bp) were loaded onto a 2,5% agarose gel and stained with SYBR^®^Safe DNA gel stain (Life Technologies).

### Generation of Expression Plasmids Coding for Patient-Derived Viral Glycoproteins

Expression plasmids coding for E1E2 from viral variants most prevalent in the patient and mouse compartments were generated. After EcoRV-digestion of the bacterial vector, the isolated DNA fragment was cloned into expression vector pcDNA3.1/Hygro (Invitrogen)(ligation using T4 DNA ligase, standard procedures). After transferring the ligation mixture into competent cells, the transformed cells were plated and the correct insert-size and orientation were confirmed by colony PCR. Positive clones were cultivated for plasmid extraction and inserts were sequenced to confirm identity.

### EIA to Measure Human Anti-E1E2 Antibody Responses

To measure E1E2 antibodies recognizing H77 and variant-specific glycoproteins, HCV_H77_ and variant-specific envelope proteins were generated and used as coating antigens in EIA. To produce glycoproteins, 293-T cells were transfected (CaCl_2_-method) with expression plasmids and the ensuing cell-lysates were used as source of E1E2 proteins. For EIA, immunoplates were coated overnight with Galanthus nivalis lectin (GNA; Sigma) and blocked with 5% Bovine Serum Albumin (BSA; Sigma). Cell-lysates were allowed to bind for 2h at room temperature. After washing, human sera were incubated and bound antibodies were visualized with anti-human IgG (Fc)-HRP (goat, Bethyl Laboratories) or anti-human IgM-HRP (goat, Sigma) conjugates and tetramethyl-benzidine (TMB) substrate. Absorbance was measured at 450 nm. For H77-specific IgG detection, sera were serially diluted to determine end-point titers, defined as the highest serum dilution giving a signal above cut-off. The EIA cut-off value (OD ≤ 0,1) was calculated by using serially diluted anti-HCV-negative sera in each assay. A positive control (HCV-positive serum with known anti-E1E2 end-point titer) was included in each assay to test the reproducibility of the assay procedure. The specificity of the EIA was further tested using anti-HCV negative controls, anti-HBsAg positive and anti-EBV positive sera. These sera never showed any reactivity. For IgG-detection to patient-derived variants, sera were assayed at a 1/100 dilution. For IgM-detection to patient-derived variants, sera were assayed at a 1/50 dilution.

### Generation of Retroviral Pseudoparticles (HCVpp), Infection and Neutralization Assay

The production of HCVpp expressing firefly luciferase and their use in neutralization assays were previously described (51). Briefly, 293T cells were co-transfected with an envelope-deficient human immunodeficiency virus (HIV) proviral genome expressing luciferase (pNL4.3.Luc.R-E-, NIH) and expression vectors encoding E1E2 glycoproteins (consensus H77c or patient-derived HCV strains), Murine Leukemia Virus (MLV) envelope or an empty vector (‘No-ENV’). HCVpp infectivity was assessed using Hep3B cells by measuring luciferase activity (Relative light units, RLU) in a luminometer (Berthold Centro LB 960). HCVpp stock dilutions resulting in ± 2x10^5^ RLU were used in neutralization assays. MLVpp and No-ENVpp served as positive and negative control in infection assays, respectively. For neutralization assays, serial two- to five-fold dilutions of sera were mixed with HCVpp, pre-incubated at 37°C for 1 hour, and added to Hep3B cells. After 72 hours, luciferase-positive cells were quantified by measuring RLU. Results are reported as IC_50_ neutralization titer, defined as the sample concentration or dilution conveying 50% reduction in the number of luciferase-positive cells.

### Statistical Methods

Mann-Whitney U tests were applied to compare continuous variables (genomic HCV-RNA in serum and B cells, E1E2 binding IgG and IgM concentrations in serum) and pairwise E1E2-sequence similarities. Fisher exact test was used to analyze 2 x 2 contingency tables. Sigmaplot 13 software (Systat Software Inc.) was used for these analyses.

### Study Approvals

Human study. Participating subjects gave written informed consent and consented to unit blood donation. The Ethical Board of the Ghent University Hospital approved the study protocol (EC # 1994/137). Animal study. The Animal Ethics Committee of the Faculty of Medicine and Health Sciences of the Ghent University approved the study protocol.

## Results

### Source of Infectious HCV in Recent and Chronic Infection

To evaluate the infectivity of HCV in recent and chronic infection we identified a cohort of 15 well-characterized patients where the duration of infection was known and clinical material available. Demographic, viral and immune characteristics of subjects with a history of IDU or blood transfusion are summarized in **Table 1**. Patients with infections of <2 years were classified as recent and those with infections of ≥2 years as chronic. Patient P15, was initially diagnosed with a genotype 3a virus (gt3a) and subsequently became infected with gt1a, donated two samples within a 3-year interval; the first in 2006 which was considered recent and the second sample in 2009 defined as chronic (**Supplementary Figure 1**). Sequencing the HCV E1E2 genes derived from serum (**Supplementary Figure 2**) enabled us to classify subjects into low (QS score <0.25), or high (QS score >0.30) complexity groups on the basis of sequence diversity (**Figure 1**). Apart from P06, an atypical chronic carrier infected with HCV gt1b for >10 years without developing nAbs, all subjects with a low QS score were recently infected with gt1a (n=4) or gt3a (n=3), whereas subjects with higher QS scores were from chronic gt1a (n=2), gt1b (n=5) and gt3a (n=1) infections.

We assessed the infectivity of sera or B cells from subjects listed in **Table 1** in human liver uPA-SCID-mice. Sera were injected *via* the peritoneal cavity (IP, containing ± 3x10^5^ IU HCV RNA) (**Figure 1**) and infection assessed by measuring peripheral viral RNA in sequential samples collected over the 8-week duration of the study. Sera from all 8 patients classified with low QS score induced an early and high-titered viremia in the mice (**Figure 1**). In contrast, sera from 6 of 8 patients with higher QS diversity did not infect the mice (**Figure 1**). The sera of patients P05 and P08, both with a high QS-score, were infectious in 1 of 3 mice tested for P05 and in 2 of 3 mice for P08. The low QS diversity pattern of donor patient sera was mirrored in the plasma of the infected mice (**Supplementary Figure 3**). Despite comparable peripheral HCV RNA levels in the low- and high QS score groups (5.4x10^5^-2.3x10^7^ and 3.7x10^5^-5.0x10^6,^ respectively) sera from recent infections were infectious, whereas sera from the chronic patients with high QS scores showed no or limited infectivity (Fisher’s exact test; p=0.007).

We hypothesized that sera from recent or acutely infected patients contained non-neutralized virions, whereas in most chronic patients HCV circulated as non-infectious immune-complexes (52, 53). To evaluate this model, we measured serum antibody responses to the HCV gt1a strain H77 E1E2 glycoproteins and assessed their ability to neutralize lentiviral pseudotypes expressing these glycoproteins (**Table 1**). Six of seven recently infected patients and the atypical chronic patient P06 had no detectable nAb responses, in contrast to the chronic patients. These data show that serum infectivity associates with the absence of circulating nAbs and is genotype-independent, supporting our model. For reasons unknown, P05 and P08 sera were infectious despite the presence of nAbs (**Table 1**). To the best of our knowledge this is the first study to evaluate the infectivity of sera from recent and chronic infected patients in uPA-SCID mice, an experiment that was previously only performed in chimpanzees (54, 55). Our study suggests that virus particles in chronic infection circulate as non-infectious immune-complexes (52, 53), which may explain their negligible infectivity.

The association between HCV infection and B cell dyscrasias (28) led us to examine the infectivity of B cells from these patients. Purified CD19^+^ B cells (~1x10^6^) were injected into the spleen of human liver chimeric uPA-SCID mice from where they can migrate directly towards the liver. B cells from 7 of 8 polyphyletic chronic patients were infectious, whereas those from all 8 patients with low QS-score failed to infect the mice (Fisher’s exact test; p=0.001) (**Figure 1**). Mice inoculated with B cells from 7 polyphyletic patients (P8-P12, P15_2009 and P16) showed evidence of circulating HCV RNA 2 weeks after inoculation (**Figure 1**). In 4 animals (K693R, K1196R, K1019 and K628R), the low viremia measured at week 2 increased over time and by week 3 was clearly positive. B cells from patient P05 failed to transmit infection. In contrast, mice receiving B cells from all 8 patients with low QS-score (7 patients and P15_2006)(**Figure 1**) showed no evidence of infection and remained HCV RNA negative until the end of follow-up (week 8).

**Figure 2:**
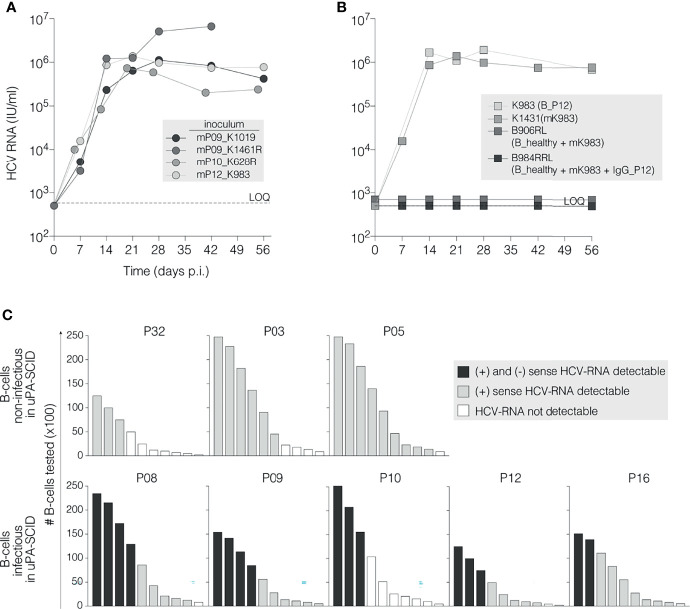
HCV RNA association with infectious B cells. **(A)** Selected virions are infectious after passage in new uPA-SCID-mice. Chimeric mice were injected with 10^4^ IU of mouse-passaged virions originating after B cell mediated HCV transmission (marked with prefix ‘m’ and ID of the originating mouse). HCV RNA levels (IU/ml) were measured in mouse plasma and plotted against time (days, X-axis). The animal injected with mP09_K1461R died before the end of the observation period. All animals demonstrate high viral replication detectable as soon as one week after viral inoculation. **(B)** Incubating B cells from a naïve donor with infectious plasma does not transmit infection. Chimeric mice were challenged (IS) with 10^6^ B cells derived from a healthy individual (B_healthy) that were pre-incubated (37°C, 2h) with 5x10^4^ IU infectious mK983 virions alone (B_healthy + mK983) or immune-complexed with 100 µg autologous IgG (B_healthy + mK983 + IgG_P12) and subsequently washed to remove non-adherent virions. As controls, the infectivity of B cells from patient P12 (B_P12) and of 10^4^ IU mouse-derived virions (mK983) is shown. Limit of HCV-RNA quantification (LOQ) is 750 IU/ml (dotted horizontal line). **(C)** Detection of (+) and (-) strand HCV RNA in infectious and non-infectious B cells. Graphic presentation of (+)ssRNA and (-)ssRNA detection in 3 non-infectious (upper panels) and 5 infectious B cells using the Tth-based RT-PCR-method as described. Bars represent the absolute amounts of B cells tested, normalized for total RNA extraction (y-axis). Progressive dilution of B cells is represented on the x-axis. Black bars refer to the simultaneous detection of (+) and (-)ssRNA; grey bars to single (+)ssRNA detection and empty bars to RNA not detectable (**Supplementary Figure 5B**).

Next, we demonstrated the infectivity of viruses in the blood of chimeric mice following B cell-delivered infection by inoculating plasma from 4 mice (K1019, K1461R, K628R and K983) into naive chimeric mice. A plasma volume containing 10^4^ IU HCV RNA was injected into the peritoneal cavity. Within one week the recipient mice became HCV RNA positive, demonstrating that B cell mediated HCV transmission generates fully infectious viral particles in a new host (**Figure 2A**). In summary, these studies demonstrate that B cells from chronic HCV patients establish productive infection in liver chimeric uPA-SCID mice, whereas B cells from recently infected patients are non-infectious.

### B Cell Mediated Infection Requires More Than Attachment of Virus to the Cell Membrane

To explore the infectious potential of the B cells, we quantified their levels of HCV RNA and matched serum samples. HCV RNA (LOQ ≥46 IU/10^6^ B cells) was detected in the purified B cells from 5 of 7 recently infected patients and from all chronic infected patients (**Table 1**). In three recently infected patients (P01, P02 and P13) no HCV RNA was detected in the B cell fraction, which may explain their non-infectious nature. Since serum HCV RNA levels in these patients (6.7x10^6^ IU/ml, 1.4x10^6^ IU/ml and 5.4x10^5^ IU/ml, respectively) were comparable to other patients, it is unlikely that B cell associated virus simply reflects the non-specific attachment of particles to the B cell surface. To experimentally test this model, we challenged chimeric mice with B cells (10^6^) purified from a non-infected individual that were pre-incubated with infectious mouse-passaged HCV (mK983, 5x10^4^ IU) alone or as an immune-complex with IgG isolated from P12. Inoculating mice with B cells from P12 or mK983 resulted in a productive infection, whereas the *ex vivo* HCV-B cell challenge samples were non-infectious. This challenge study suggests that successful B cell mediated infection requires more than just attachment of HCV to the B cell surface (**Figure 2B**). To evaluate whether B cells from patients with chronic infection support some level of HCV replication we quantified the replicative intermediate negative strand RNA using a previously reported strand-specific RT-PCR protocol (48) (**Supplementary Figure 5**). No positive or negative strand RNA was detected in B cells from non-viral infected healthy donors, validating the specificity of our PCR method. **Figure 2C** shows the presence of positive strand RNA in B cells purified from 8 patients (two recent (P03 and P32) and 5 chronic infections (P8-P12 and P16) and atypical chronic patient P05). Negative strand RNA was only detected in B cells from the chronic patients that were infectious for the mice (**Figure 2C**), consistent with B cells supporting modest HCV replication *in vivo* during chronic infection.

### Quasispecies Analysis of B Cell Mediated Infection

**Figure 3:**
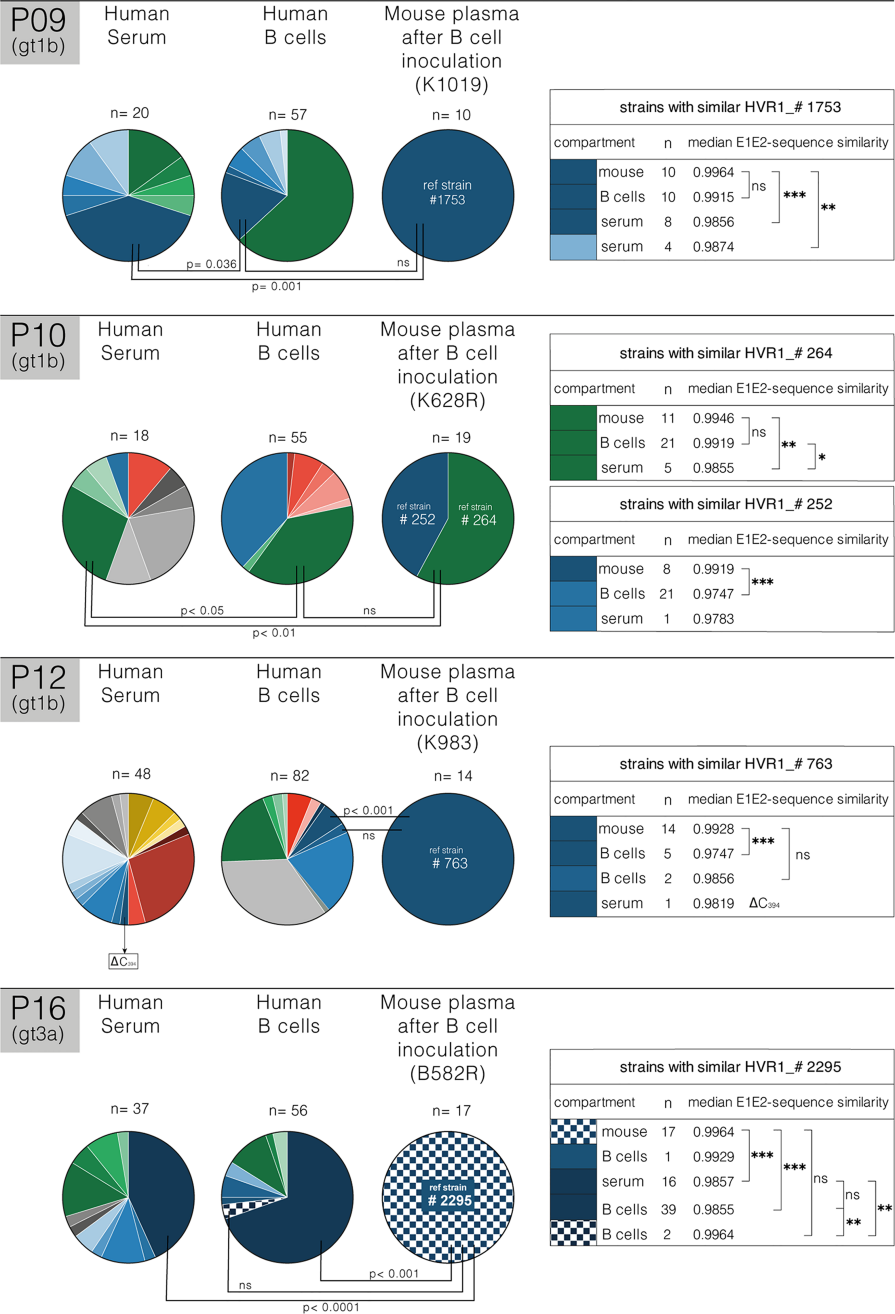
Quasispecies analysis of B cell mediated infection. Schematic representation of HVR1 viral quasispecies (QS) distribution in human serum and B cells at the time of injection and in uPA-SCID plasma for three gt1b-patients (P09, P10 and P12) and one gt3a-patient (P16). Pie charts represent the fraction of each clone per compartment based on HVR1-diversity (number of clones analyzed, and mouse identification are shown). HVR1-isolates are unique for each patient and indicated by different colors. Closely related HVR1-variants, based on individual AA-similarity and clustering in phylogenetic analysis, are depicted in different intensities of the same color (**Supplementary Figure 4**). For each patient a representative mouse strain (#1753,…) is depicted and pairwise full E1E2 similarity to similar strains in other compartments was calculated using the IDENTIFY similarity matrix for pairwise alignment (Bioedit Sequence Alignment Editor, right panel). For each compartment, the number (n) of similar clones and their median E1E2-sequence similarity with mouse-derived strains are shown in the tables on the right. Differences between pairwise E1E2-sequence similarities were calculated (*p<0.05; **p<0.01; ***p<0.001; ns, non-significant; Mann-Whitney U test) and shown on the pie charts. In the serum of P12, the only strain that showed similarities to the reference mouse strain carried a C-deletion at position AA_394_ (Δ C_394_), suggesting non-viability. For P16, variability in two additional E2-regions was considered and depicted by shading. Analysis demonstrates that mouse-derived strains occurring after B cell transfer closely resemble strains found in the B cell compartment (p=ns), and are significantly different from serum-derived strains.

To elucidate the origin of the B cell transmitted virus, we compared the HCV QS in the human serum and B cell inocula with the plasma from the infected mice. **Figure 3** shows this comparative analysis for chronic patients P09, P10, P12 and P16. Viral sequences were PCR amplified from each compartment (serum, B cell and mouse plasma) and E1E2 variability assessed. The genetic diversity of the first hypervariable region (HVR1) of viruses associated with B cells was substantial and largely reflected that found in serum, and in all patients, compartmentalization of variants in B cells was observed. However, in P12 several variants (marked in green) were identified in the B cell fraction that did not occur in serum. As P12 is a former IDU, these variants could have been acquired through super-infection, however, this is inconsistent with our phylogenetic analyses that shows all variants forming a single cluster (**Supplementary Figure 2**). After successful B cell mediated infection of the mice, QS analyses of the plasma showed a limited viral diversity. Given the limited immune pressure in the chimeric uPA-SCID mice, these variants most likely reflect the infecting inoculum and support a model where infection is initiated by a limited number of founder viruses. For each patient, a representative strain derived from the mouse compartment was identified and pairwise amino acid-similarity to strains in other compartments calculated (IDENTIFY similarity matrix). For P09, clones with identical HVR1 to #1753 (mouse reference strain) were found in the B cell (10 clones) and serum (8 clones) compartment. Pairwise E1E2 alignment revealed that clones from the infected mice most closely resemble variants from the B cells and differed significantly from serum variants (p=0.001). We noted a similar observation for P10, with mouse plasma variants (reference strains #264) more closely resembling B cell variants than the serum (p<0.01). The origin of reference strain #252 variants could not be elucidated. For P12, 5 clones with identical HVR1 to #763 (mouse reference strain) were found in the B cell compartment, but these clones differ significantly from the mouse strains at the E1E2 level (p<0.001). However, two clones with a nearly identical HVR1 to #763 were found in the B cell compartment that closely resembled the mouse strain variants at the E1E2-level (p=ns). Furthermore, the only strain that showed similarities to the reference mouse strain in the serum of P12 carried a C-deletion at amino acid position 394, suggesting non-viability. For P16, a clone with identical HVR1 and similar E1E2 (0.9929) to #2295 (mouse reference strain) was only found in the B cell compartment. Two other clones in the B cell compartment more closely resemble (0.9964) reference strain #2295 at the E1E2-level. Compared to the mouse strains, these clones are nearly identical in HVR1 and identical in two other E2-regions (depicted by shading). Overall, pairwise comparisons of full E1E2 amino acid sequences of HCV present in human serum and B cells and mouse plasma demonstrated that viral strains isolated from mouse plasma after B cell transfer closely resembled at least one strain found in the B cell compartment while they differed significantly from serum-derived strains.

### HCV Glycoproteins Associated With B Cells Are Recognized by IgM

**Figure 4:**
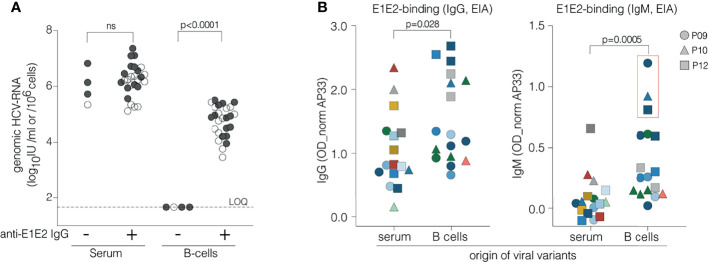
Relationship between circulating IgG, IgM, B cell associated HCV RNA and viral glycoprotein recognition. **(A)** Association between circulating anti-E1E2 IgG and genomic HCV RNA in serum and B cells. Anti-E1E2 IgG was measured in serum and genomic HCV RNA in serum and B cells from 29 HCV patients, 15 studied in detail herein (P15 in 2006 and 2009) (●) and 14 additional ones (○). Patients are grouped according to absence (4 patients) or presence (26 specimens from 25 patients) of anti-E1E2 IgG in serum and occurrence of genomic HCV RNA in serum and B cells is examined in both groups. HCV RNA is present in all sera irrespective of circulating anti-E1E2 IgG whereas it is only detected in B cells of patients with serum anti-E1E2 IgG. **(B)** Binding of IgG and IgM to E1E2 proteins derived from different compartments. E1E2-proteins were generated using cDNA clones of the most prevalent variants isolated from serum and B cells from patients P09 (●), P10 (▲) and P12 (■). Each symbol represents a unique E1E2-protein while its color refers to the strain of origin as shown in **Figure 3** and **Supplementary Figure 4**. Serum from patients P09, P10 and P12 was assayed at dilutions 1/100 (IgG) or 1/50 (IgM) in an EIA with GNA-captured E1E2 proteins. E1E2-specific IgG binds better to envelope proteins derived from B cells than to serum variants (p=0.028, Mann-Whitney U test). This preference is even more pronounced in E1E2-specific IgM (p=0.0005, Mann-Whitney U test) that shows the strongest binding with proteins representing the viral variants isolated from B cells shown to be closely related to isolates observed in plasma of B cell infected mice (shown in the red box). ns, not significant.

The data summarized in **Table 1** suggest a possible association between serum anti-HCV E1E2 IgG and B cell associated genomic RNA. To evaluate this further we measured anti-HCV E1E2 IgG levels in the serum and HCV RNA levels in the periphery and associated with B cells from 29 HCV infected patients (one acute and 13 chronic patients in addition to those listed in **Table 1**, where P15 was sampled in 2006 and 2009). Peripheral HCV RNA levels in the sera ranged from 1x10^5^ to 2.3x10^7^ IU/ml, whereas B cell associated HCV RNA was only found in patients with detectable anti-HCV E1E2 IgG (**Figure 4A**). To evaluate the potential role of viral specific IgM and IgG in this process, antibody responses against endogenous viral glycoprotein variants cloned from serum and B cells from patients P09, P10 and P12 were examined (**Figure 4B**). E1E2-proteins representing the predominant variants in serum and B cells from these three patients were generated and IgG- and IgM binding of these variants was evaluated. IgG-recognition of viral variants was diverse and not restricted to their originating compartments (**Figure 4B**, left panel). IgM antibodies instead preferentially recognized variants originating from B cells that closely resembled those isolated from infected mouse plasma (**Figure 4B**, right panel), suggesting a role for IgM in binding B cell associated HCV.

## Discussion

Our study shows the differential infectivity of HCV for humanized uPA-SCID mice in samples from acute and chronic stages of infection. Peripheral circulating virus from recently infected patients was infectious, whereas sera from most chronic patients were non-infectious for the mice. Importantly, CD19^+^ B cells from chronic patients harboring highly diverse quasispecies established infection in the mice whereas B cells from recently infected patients were non-infectious; highlighting a dichotomy in the infectious source of HCV. In addition to the numerous variants that we observed in serum, B cells carried virus that most closely resembled virus isolated from mice infected *via* B cells. Negative strand viral RNA was only detected in B cells capable of transmitting infection. Furthermore, B cells harboring viral RNA were predominantly found in patients with circulating antibodies specific for HCV E1E2 glycoproteins. IgM rather than IgG antibodies preferentially recognized variants originating from B cells, suggesting a role for IgM in mediating HCV association with B cells.

Our understanding of the parameters defining infectious HCV in humans is incomplete due to the limited replication of primary HCV strains in any *in vitro* model system (56, 57). Previous studies have reported on the infectivity of patient-derived sera in chimpanzees (34) and humanized uPA-SCID mice (42, 54, 58), however, these studies were limited by the small number of clinical samples. Here we demonstrate that serum infectivity associates with the short duration of infection and absence of circulating nAbs. Of the 7 patients with recent infections, three had no detectable anti-envelope Abs and 6 had no circulating nAbs. In contrast, sera from all chronic patients but one (P06) contained anti-E1E2 Abs with neutralizing capacity. We hypothesize that the infectious sera from recently infected patients contains non-neutralized virions, whereas in chronic disease the virus circulates as non-infectious immune-complexes (52, 53). These results, together with reports that HCV infection of chimeric liver mouse models can be neutralized by the passive transfer of monoclonal antibodies targeting the HCV-E2 glycoprotein, provides optimism on the feasibility of effective HCV vaccines (20, 22, 59, 60). Our observation that B cell associated HCV RNA is only observed in patients with circulating anti-E1E2 IgG suggests a possible link between envelope-specific Abs and/or the B cells expressing these antibodies in transmitting HCV. Our data suggest that the presence of envelope-specific IgG and B cell-associated HCV RNA is not sufficient for B cell mediated infection. Analyses of positive and negative strand HCV RNA in B cells from 8 patients showed that B cell mediated infection associates with the presence of negative strand RNA, suggesting a low level of replication in these cells.

Analyzing HCV envelope specific antibodies showed that viral glycoproteins originating from B cells that were infectious for the mice were preferentially recognized by IgM antibodies. One possible scenario is that HCV variants originating in the liver for which there is no IgG response can associate with the IgM-B cell receptor (BCR) on circulating or intrahepatic B cells. In human peripheral blood, the predominant B cell populations are naive IgM^+^CD27^−^ B cells, with non-mutated immunoglobulin genes, and class-switched memory IgG^+^CD27^+^ B cells. Recently, a population of IgM^+^CD27^+^ memory B cells, were identified which can re-enter germinal center reactions and differentiate into antibody-secreting cells (61–63), however the generation and immune function of these cells is poorly understood. Kong et al. (64) reported a higher frequency of this IgM^+^CD27^+^ memory B cell subset in chronic hepatitis C compared to healthy controls. Tucci et al. (65) reported the presence of IgM^+^CD27^+^ B cell clones in chronic hepatitis C. Despite several attempts we were unable to isolate sufficient IgM^+^CD27^+^ B cells from our cohort of patients to demonstrate an enrichment of HCV RNA in this subpopulation. The expression of CXCR3, an inflamed tissue homing receptor, and CD11c on B cells enables these cells to home to the inflamed liver, where CXCR3-ligands are expressed (66). Reports showing the increased expression of CXCR3^+^ on CD27^+^ B cells in chronic hepatitis C (67–69) support a model where these B cells could bind HCV and trans-infect permissive hepatocytes in the liver. This model predicts that nAbs induced by vaccination or natural infection may be unable to prevent HCV infection or reinfection when the virus is transmitted *via* B cells. Due to a lack of appropriate biological samples, we were unable to test this hypothesis by challenging passively vaccinated mice with B cells from a chronic patient. Our observation that envelope-specific antibodies are associated with B cell mediated viral transmission has important implications. The occurrence of envelope-specific nAbs is a hallmark of chronicity and the evolution of the B cell repertoire. Gp-specific IgM, in particular directed against novel variants, may be a biomarker for the presence of HCV transmitting B cells rather than being itself the mediator of transmission. The potential role of B cells in chronic HCV infection warrants further research to understand the etiology of B cell lymphoproliferative disorders and viral persistence.

## Data Availability Statement

The original contributions presented in the study are included in the article/**Supplementary Material**. Further inquiries can be directed to the corresponding author.

## Ethics Statement

The studies involving human participants were reviewed and approved by Ethical Board of the Ghent University Hospital. The patients/participants provided their written informed consent to participate in this study. The animal study was reviewed and approved by The Animal Ethics Committee, Faculty of Medicine and Health Sciences, Ghent University.

## Author Contributions

Study conception and design: ID, PM, and GL-R. Acquisition and data analysis: ID, FH, LV, AF, CB, YG, SC, ZS, WS, AE, AM, HV, PM, JM, and GL-R. Analysis and interpretation of data: ID, JM, PM, and GL-R. Drafting of manuscript: ID, JM, and GL-R. All authors have critically revised the manuscript and approved the final version.

## Funding

The authors acknowledge financial support of their work by the Research Foundation Flanders (FWO project G.0212.N), the Ghent University (GOA_01G01712), the City of Ghent and the European Union (FP7_HepaMab). JM laboratory is funded by Wellcome Trust IA 200838/Z/16/Z and MRC project grant MR/R022011/1.

## Conflict of Interest

The authors declare that the research was conducted in the absence of any commercial or financial relationships that could be construed as a potential conflict of interest.

## Publisher’s Note

All claims expressed in this article are solely those of the authors and do not necessarily represent those of their affiliated organizations, or those of the publisher, the editors and the reviewers. Any product that may be evaluated in this article, or claim that may be made by its manufacturer, is not guaranteed or endorsed by the publisher.
