# Supplementary material for: A Role for B Cells to Transmit Hepatitis C Virus Infection
Source: Front Immunol. 2021 Dec 16;12:775098. doi: 10.3389/fimmu.2021.775098 (PMC8716873; doi:10.3389/fimmu.2021.775098)
Supplement: Supplementary file 1 [file DataSheet_1.docx]

Supplementary Material

Supplementary Figure 1

**A**

**B**

**Supplementary Figure 1. Virologic and immunologic characteristics of Patient 15 who experienced a HCV-superinfection. (A)** Serum and B cells, examined in the present study, have been taken in 2006 and 2009 and are shown as P15_2006 and P15_2009 (shaded areas). HCV-genotyping of human sera was performed using a Line Probe Assay (LiPA) developed by Innogenetics (Versant® HCV genotype Assay) and by genotype specific PCR-amplification of the E1E2 region (*in house*). The patient reported intravenous drug use at the time of superinfection. **(B)** Evolution of E1E2-specific immunological responses since time of initial viremia. Anti-E1E2_H77_ IgG and anti-E1E2_S52_ IgG levels are expressed as reciprocal EIA-titer. Neutralizing responses of autologous serum to HCV-pseudoviral particles expressing envelope proteins of gt1a (HCVpp_H77) or gt3a (HCVpp_S52) are expressed as IC_50_ (reciprocal titer).

**Supplementary Figure 2**

**
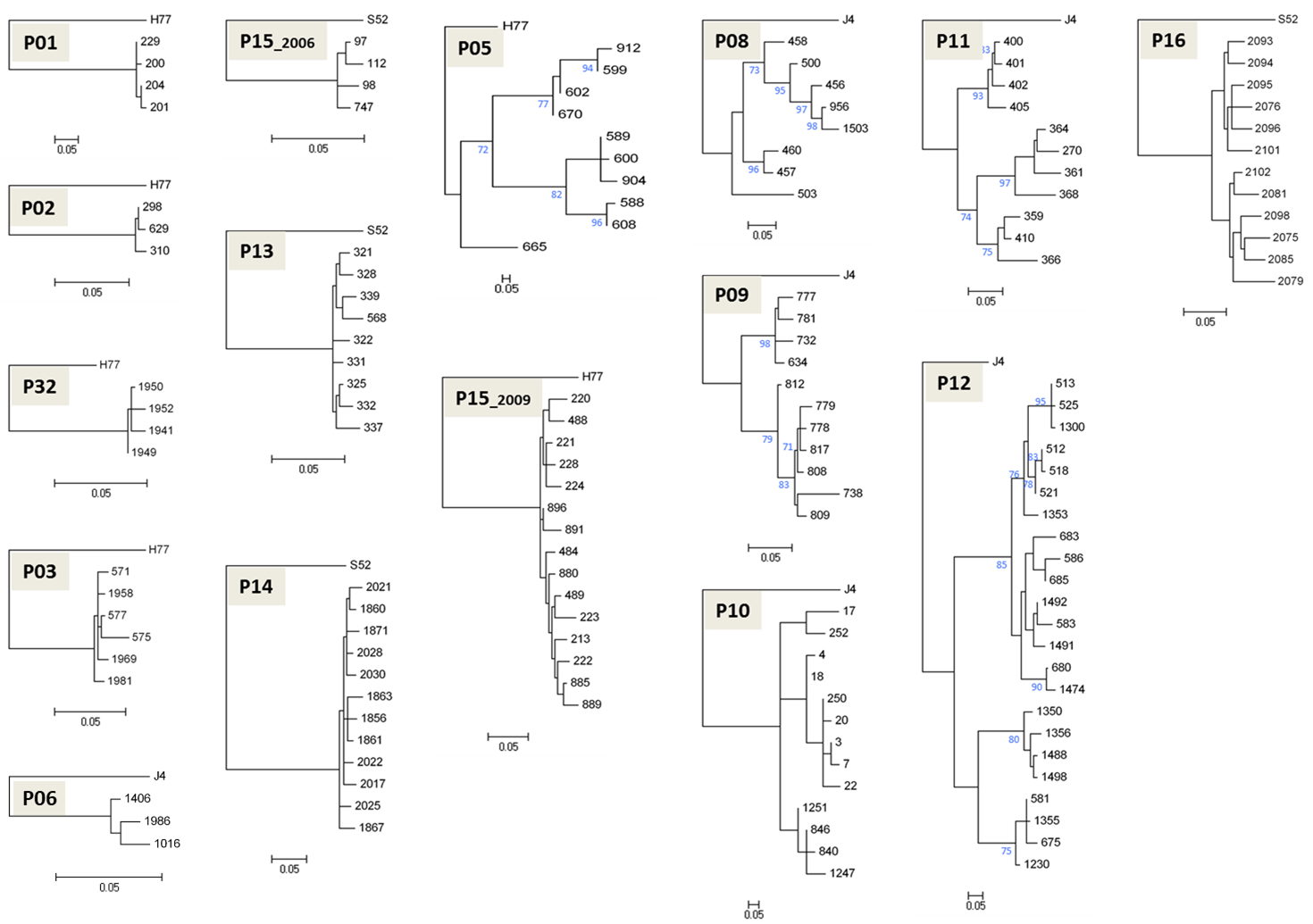
**

**Supplementary Figure 2. Phylogenetic analyses of HCV-infected patients.** Maximum-Likelihood phylogenetic trees showing the relationship between E1E2_192-745_ sequences of the major viral variants were inferred under the LG model of AA substitution with estimated γ- distribution parameter, using PhyML software (v3.0; South of France bioinformatics platform; ATGC). The tree topology (starting with a Neighbor-Joining tree) was optimized using the Nearest Neighbor Interchange (NNI) approach. Bootstrapping with 1000 replicates tested the statistical robustness of the phylogenetic trees. Only branching with a bootstrap value of ≥70% was defined as robust clustering (depicted in blue). Phylogenetic trees are rooted on the consensus sequence for each genotype (HCV-H77 for gt1a, HCV-J4 for gt1b and HCV-S52 for gt3a). For each tree, the black numbers (tree-leaves) are clone-codes, each corresponding to a unique E1E2-AA sequence. An estimate of the genetic distance is presented as a bar at the bottom of each tree.

**Supplementary Figure 3.**

**Supplementary Figure 3. HVR1-diversity in human serum and in uPA-SCID mice plasma following inoculation with serum from patients with low QS-diversity.** Schematic representation of the E1E2-HVR1 viral quasispecies (QS) distribution in human serum and mouse plasma for four gt1a-patients (P01, P02, P32 and P03), one gt1b-patient (P06) and one gt3a-patient (P15_2006) with QS-score <0.2 (Figure 1, Supplementary Figure 2). The number of clones analysed per compartment is shown beneath each pie chart that represents the fraction of each clone/compartment based on diversity in HVR1. Based on individual AA-similarity and clustering in phylogenetic analysis, all HVR1-sequences shown are closely related within each patient.

**Supplementary Figure 4.**


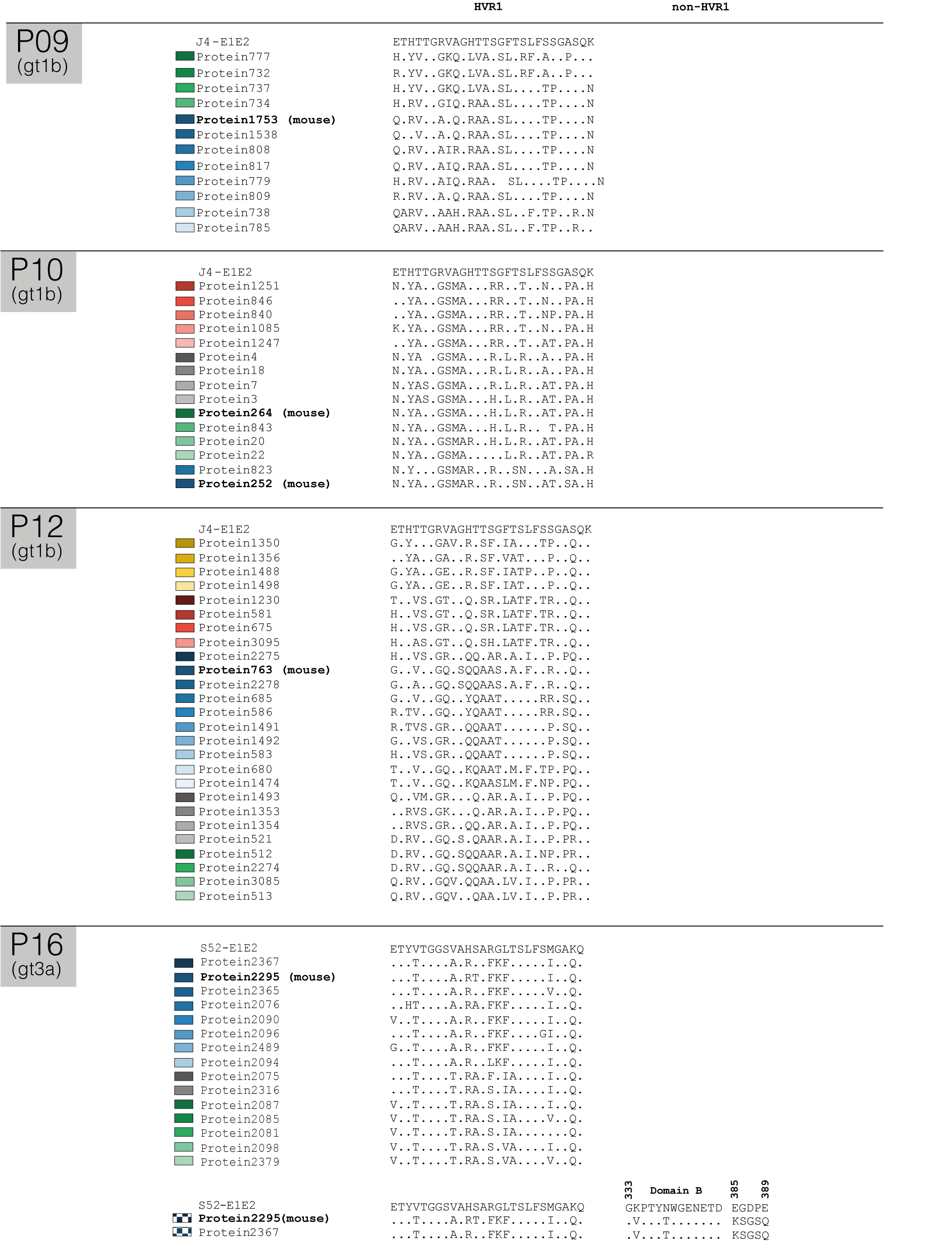


**Supplementary Figure 4. Amino acid sequences and corresponding colour codes of viral variants (HVR1) depicted in Figure 3**. Schematic representation of the E1E2-HVR1 viral quasispecies (QS) distribution in human serum and B cells at the time of injection and in uPA-SCID plasma for three gt1b-patients (P09, P10 and P12) and one gt3a-patient (P16) is shown in Figure 3. Viral HVR1-isolates are indicated by different colours and are unique for each patient. The AA sequences of the HVR1 region (AA_384-410_) and their corresponding colour codes are shown. The consensus sequences HCV-J4 (gt1b) and HCV-S52 (gt3a) are used for HVR1-alignments and shown on top. Closely related HVR1-variants (based on individual AA-similarity and clustering in phylogenetic analysis) are depicted in different intensities of the same colour.

**Supplementary Figure 5**

**A**

**
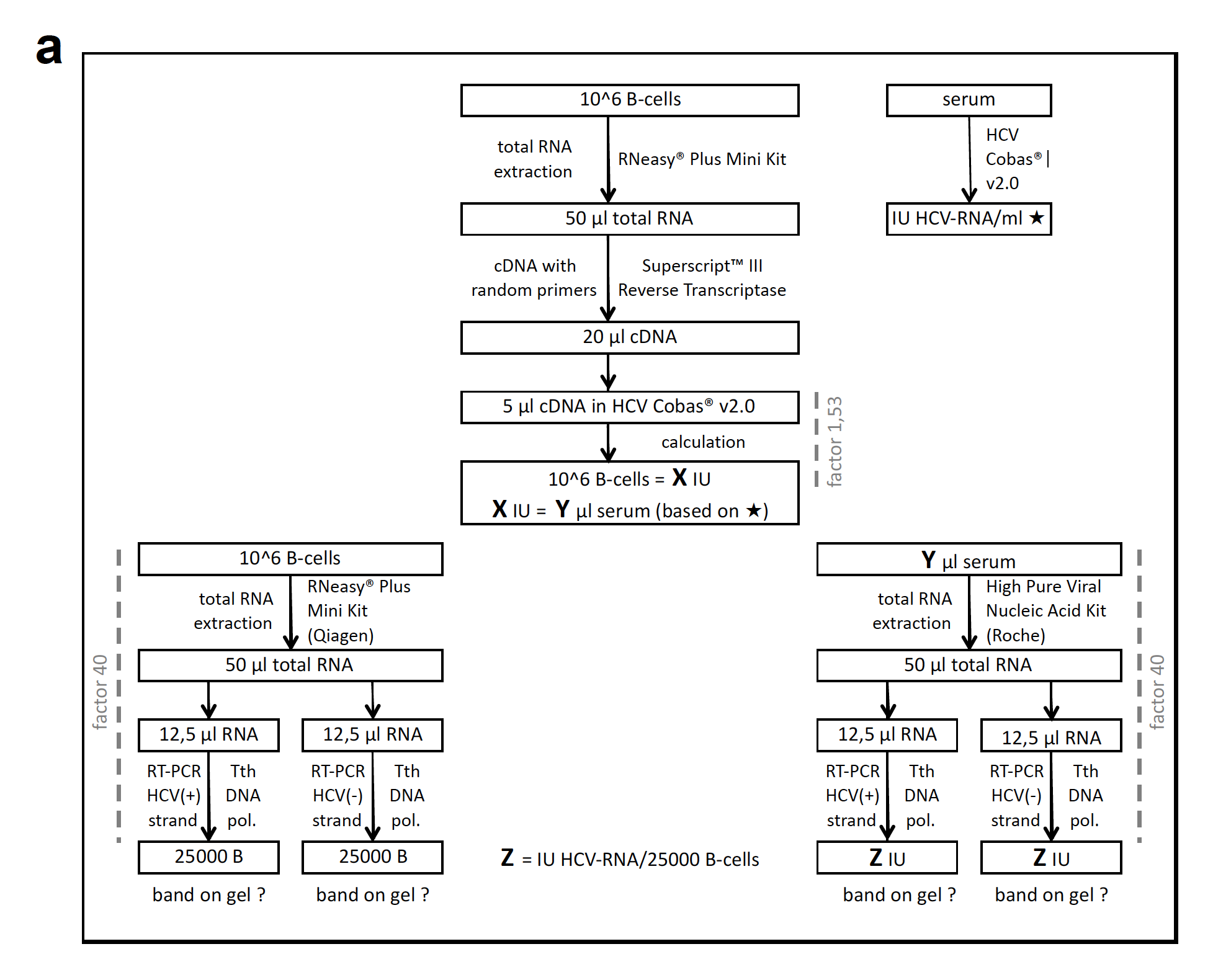
**

**B**
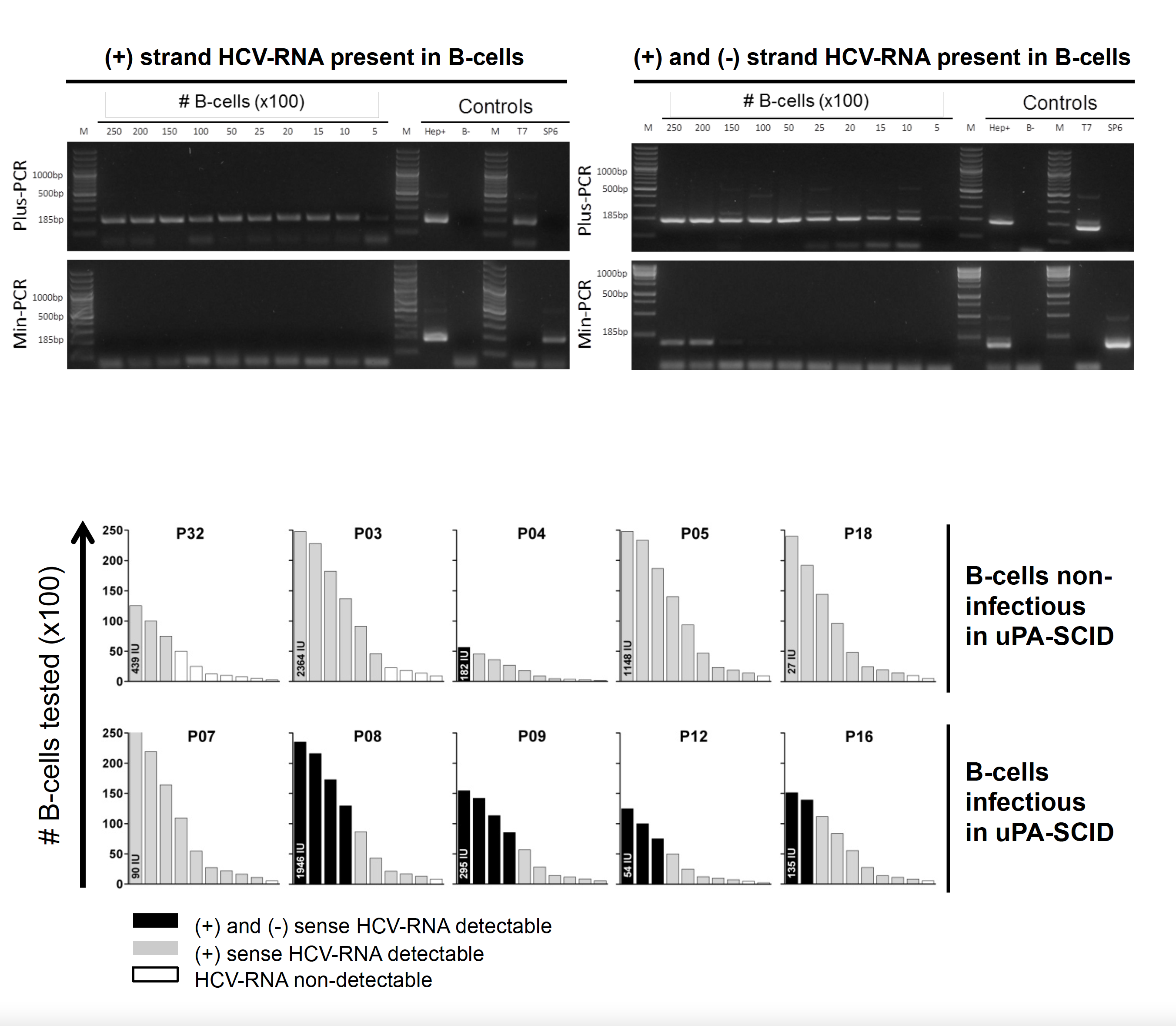


**Supplementary Figure 5. Presence of (+) and (-) single strand HCV-RNA in infectious and non-infectious B cells.** (**A**) Flow chart illustrates the sequence of basic steps in (i) the quantification of HCV-RNA in B cells using the COBAS® TaqMan® HCV Test v2.0 (Roche) and (ii) the detection of (+)ssRNA and (-)ssRNA in B cells and serum using a strand-specific RT-PCR based on Tth DNA polymerase (detailed in methods). (**B**) Representative example of an agarose gel electrophoresis showing the semi-quantitative detection of (+)ssRNA (Plus-PCR, upper part of both left and right panels) and (-)ssRNA (Min-PCR, lower part of both left and right panels) in non-infectious B cells (P05, HCV-gt1a) and infectious B cells (P08, HCV-gt1b). After total RNA extraction from B cells, (-) and (+) strand-specific cDNA was generated and amplified using a Tth-based RT-PCR-protocol. The presence of ssRNA was monitored on a dilution series representing absolute amounts of B cells (normalized for total RNA content after extraction). HCV_JC1-infected Huh7.5 hepatoma-cells (referred as **Hep+**) and B cells from non-infected individuals (referred as **B-**) were used as HCV-positive and -negative control cells, respectively. Synthesized appropriate positive- (**T7**) and negative-strand (**SP6**) HCV-RNA control fragments were added in each run (kindly provided by Harvey Alter, NIH). M, 100 bp DNA ladder

**Supplementary Table 1.**

^a^IDU, injecting drug use. ^b^Time elapsed between initial HCV-infection/diagnosis and biological sampling for the present study (y, year; m, month). ^c^Anti-E1E2_H77_ IgG levels are expressed as reciprocal endpoint dilution titer of autologous serum in EIA; <10, IgG was not detected at serum-dilution 1/10. ^d^Neutralizing activities of autologous serum to HCV-pseudoviral particles expressing envelope proteins of gt1a (HCVpp_H77) are expressed as IC_50_ (reciprocal titer); <50%, neutralization did not reach 50%.

**Supplementary Table 2.**

|  |  | **serum injection** | |  | **B cell injection** | |  | **% CD19+ cells** | |
| --- | --- | --- | --- | --- | --- | --- | --- | --- | --- |
|  | Patient ID | mouse ID | hu-Alb (mg/ml) |  | mouse ID | hu-Alb (mg/ml) |  | PBMC | after purification (=injected) |
| Figure 1 | P01 | K753 | 5.6 |  | K750L | 5.8 |  | 6 | 96 |
|  | P02 | K874 | 8.7 |  | B133L | 5.1 |  | 12 | 98 |
|  | P32 | B582 | 3.3 |  | K874R | 4.0 |  | 19 | 99 |
|  | P03 | B629L | 4.1 |  | K813R | 3.9 |  | 12 | 98 |
|  |  |  |  |  | B147L | 6.2 |  | 13 | 99 |
|  | P06 | K594 | 2.9 |  | K628 | 2.7 |  | 14 | 98 |
|  | P15_2006 | B78R | 4.4 |  | K874RL | 2.4 |  | 22 | 99 |
|  | P13 | B160 | 4.8 |  | K884 | 6.9 |  | 8 | 98 |
|  | P14 | K993 | 10.2 |  | B673RL | 4.8 |  | 13 | 99 |
|  | P05 | B295R | 3.8 |  | K687R | 4.3 |  | 22 | 98 |
|  |  | B682 | 3.7 |  | K1030 | 9.0 |  | 18 | 99 |
|  |  | B507 | 4.9 |  | K1290 | 5.0 |  | 20 | 98 |
|  | P15_2009 | B85L | 4.0 |  | K811 | 9.6 |  | 18 | 98 |
|  |  | B828L | 4.7 |  |  |  |  |  |  |
|  | P08 | K738 | 2.0 |  | K693R | 2.1 |  | 15 | 99 |
|  |  | B259L | 4.8 |  | B186R | 4.4 |  | 13 | 99 |
|  |  | K1197L | 6.3 |  | K1196R | 6.1 |  | 14 | 99 |
|  | P09 | K1045R | 6.8 |  | K1019 | 3.6 |  | 29 | 98 |
|  |  | B528 | 8.5 |  | B720RL | 6.9 |  | 27 | 99 |
|  |  |  |  |  | K1461R | 8.2 |  | 30 | 99 |
|  | P10 | K579 | 3.4 |  | K628R | 2.0 |  | 6 | 98 |
|  |  | B505RL | 4.9 |  |  |  |  |  |  |
|  |  | B528R | 6.5 |  |  |  |  |  |  |
|  | P11 | K829L | 2.1 |  | K797 | 3.3 |  | 26 | 98 |
|  |  | K1190R | 4.0 |  |  |  |  |  |  |
|  | P12 | K1106L | 4.5 |  | K983 | 3.6 |  | 16 | 98 |
|  |  |  |  |  | K1772R | 5.2 |  | 17 | 99 |
|  | P16 | B584R | 3.1 |  | B582R | 4.8 |  | 12 | 98 |
| Figure 2A | mP09_K1019 |  | 3.4 |  |  |  |  |  |  |
|  | mP09_K1461R |  | 3.9 |  |  |  |  |  |  |
|  | mP10_K628R |  | 5.4 |  |  |  |  |  |  |
|  | mP12_K983 |  | 5.0 |  |  |  |  |  |  |
| Figure 2B | B_healthy + mK983 |  |  |  |  | 4.5 |  |  |  |
|  | B_healthy + mK983 + IgG_P12 |  |  |  |  | 4.0 |  |  |  |
